# Combined detection of HLA-B27, ESR, and CRP as biochemical and immunogenetic markers in the auxiliary diagnosis of ankylosing spondylitis

**DOI:** 10.5937/jomb0-61518

**Published:** 2026-02-27

**Authors:** Muchuan Wang, Siyuan Yao, Chengao Gao, Yipeng Wang

**Affiliations:** 1 Capital Medical University, Beijing Jishuitan Hospital, Department of Sports Medicine, Beijing, China; 2 Beijing Shijitan Hospital Affiliated to Capital Medical University, Department of Spine Surgery, Beijing, China; 3 Peking Union Medical College Hospital, Department of Orthopedic Surgery, Beijing, China

**Keywords:** Ankylosing spondylitis, HLA-B27, erythrocyte sedimentation rate, C-reactive protein, biochemical markers, laboratory diagnosis, Ankilozni spondilitis, HLA-B27, brzina sedimentacije eritrocita, C-reaktivni protein, biohemijski markeri, laboratorijska dijagnoza

## Abstract

**Background:**

Ankylosing spondylitis (AS) is a chronic inflammatory disease in which early diagnosis is often challenging due to atypical clinical manifestations. Laboratory biomarkers such as human leukocyte antigen-B27 (HLA-B27), erythrocyte sedimentation rate (ESR), and C-reactive protein (CRP) play an essential role in diagnostic evaluation.

**Methods:**

A total of 120 AS patients and 100 healthy controls were enrolled. Venous blood samples were analyzed for HLA-B27 (flow cytometry), ESR (Westergren method), and CRP (immunoturbidimetry). The Bath Ankylosing Spondylitis Disease Activity Index (BASDAI) was assessed, and diagnostic performance was evaluated using receiver operating characteristic (ROC) curve analysis.

**Results:**

HLA-B27 positivity was significantly higher in the AS group than in controls (92.5% vs. 5.0%, P&lt; 0.001). Both ESR and CRP levels were markedly elevated in AS patients and showed a progressive increase with higher BASDAI scores (P&lt; 0.05). ROC analysis demonstrated superior diagnostic efficacy of combined HLA-B27, ESR, and CRP detection (AUC=1.000, sensitivity 97.2%, specificity 93.3%) compared with single markers.

**Conclusions:**

The combined detection of HLA-B27, ESR, and CRP provides a reliable laboratory-based diagnostic approach for ankylosing spondylitis, enhancing accuracy and clinical applicability. These findings highlight the importance of integrating biochemical and immunogenetic markers into routine diagnostic practice.

## Abstract (sr)

**Uvod:**

Ankilozni spondilitis (AS) je hronična inflamatorna bolest kod koje je rana dijagnoza često teska zbog atipičnih kliničkih manifestacija. Laboratorijski biomarkeri kao sto su humani leukocitarni antigen-B27 (HLA-B27), brzina sedimentacije eritrocita (SE) i C-reaktivni protein (CRP) igraju sustinsku ulogu u dijagnostičkoj proceni.

**Metode:**

Ukupno je uključeno 120 pacijenata sa AS i 100 zdravih kontrola. Uzorci venske krvi analizirani su na HLA-B27 (protočna citometrija), SE (Vesterg renova metoda) i CRP (imunoturbidimetrija). Procenjen je indeks aktivnosti ankilozirajućeg spondilitisa u Batu (BASDAI), a dijagnostičke performanse su procenjene korišćenjem analize ROC krive.

**Rezultati:**

Pozitivnost HLA-B27 bila je značajno veća u grupi sa AS nego u kontrolnoj grupi (92,5% naspram 5,0%, P&lt;0,001). Nivoi i sedimentacije eritrocita (ESR) i CRP bili su značajno poviseni kod pacijenata sa AS i pokazali su progresivan porast sa višim BASDAI rezultatima (P&lt;0,05). ROC analiza je pokazala superiornu dijagnostičku efikasnost kombinovane detekcije HLA-B27, SER i CRP (AUC=1,000, osetljivost 97,2%, specifičnost 93,3%) u poređenju sa pojedinačnim markerima.

**Zaključak:**

Kombinovana detekcija HLA-B27, sedimentacije eritrocita (ESR) i CRP pruža pouzdan laboratorijski dijagnostički pristup za ankilozni spondilitis, povećavajući tačnost i kliničku primenljivost. Ovi nalazi ističu važnost integracije biohemijskih i imunogenetskih markera u rutinsku dijagnostičku praksu.

## Introduction

Ankylosing spondylitis (AS) is a chronic inflammatory disease that markedly impairs patients' quality of life and physical function [1][2]. Its insidious onset and nonspecific early manifestations often lead to misdiagnosis or delayed diagnosis, postponing timely treatment [3][4]. Consequently, identifying reliable auxiliary diagnostic methods is crucial for improving early recognition and management of AS.

Among laboratory parameters, human leukocyte antigen B27 (HLA B27), erythrocyte sedimentation rate (ESR), and C-reactive protein (CRP) have demonstrated substantial diagnostic utility in AS. HLA B27, a major histocompatibility complex class I molecule, is strongly associated with disease susceptibility, with positivity rates reported in up to 90% of patients [5]. Its detection provides important diagnostic clues, particularly in individuals with atypical clinical presentations. ESR and CRP - classical biochemical markers of systemic inflammation - are frequently elevated in AS and can dynamically reflect disease activity and therapeutic response, typically rising during active phases and declining following effective anti-inflammatory treatment.

Nevertheless, the diagnostic use of single biomarkers has limitations. HLA-B27 positivity is not exclusive to AS and can be detected in other diseases or even among healthy individuals, while elevated ESR and CRP lack disease specificity and occur in a broad spectrum of inflammatory disorders [6][7]. Therefore, combining immunogenetic (HLA-B27) with biochemical (ESR, CRP) parameters may enhance diagnostic accuracy and sensitivity, offering a more comprehensive and reliable laboratory approach for AS diagnosis [8].

The present study aimed to evaluate the clinical value of combined detection of HLA-B27, ESR, and CRP in the auxiliary diagnosis of AS, thereby providing evidence for their integration into routine laboratory practice.

## Materials and methods

### Study population

From June 2023 to February 2025, a total of 120 patients diagnosed with ankylosing spondylitis (AS) were recruited from our hospital and assigned to the case group. Additionally, 100 age- and sex-matched healthy individuals undergoing routine physical examinations during the same period were enrolled as the control group. Among AS patients, 85 were male (70.8%) and 35 female (29.2%), aged 18-65 years (mean 32.5 ± 8.7 years). The control group included 68 males (68.0%) and 32 females (32.0%), aged 20-64 years (mean 31.8 ± 9.1 years). No significant differences in sex or age distribution were observed between the two groups (*P*>0.05).

### Inclusion and exclusion criteria

Participants were eligible if they were 18-65 years old, met established diagnostic criteria for ankylosing spondylitis, and had complete clinical and laboratory records. Exclusion criteria included other rheumatic immune diseases (e.g., rheumatoid arthritis, psoriatic arthritis); concurrent infections, malignancies, or other conditions that could affect biomarker measurements; receipt of systemic corticosteroids or immunosuppressants within the past month; and pregnancy or lactation.

### Laboratory measurements

Fasting venous blood (5 mL) was collected from each participant and divided for analysis of three laboratory parameters: 1) HLA-B27 detection: Flow cytometry was performed using a BD FACS Canto II cytometer (BD Biosciences, USA) with PE-conjugated anti-HLA-B27 monoclonal antibody (catalog no. 555554, BD Biosciences); 2) ESR measurement: Conducted by the Westergren method using an ESR-30 automated analyzer (Medonic, Germany); 3) CRP determination: Assessed by immunoturbidimetry on a Hitachi 7600 automatic biochemical analyzer (Hitachi, Japan). CRP assay kits were supplied by Shanghai Kehua Bioengineering Co., Ltd.

All procedures strictly followed manufacturer instructions. Analyses were performed independently by two experienced laboratory technologists, and the mean value was used for evaluation.

Disease activity in the AS group was assessed by the Bath Ankylosing Spondylitis Disease Activity Index (BASDAI). Scores of 0-4 indicated mild activity, 4-7 moderate activity, and 7-10 severe activity.

### Statistical analysis

Data were double-entered and stored in Excel, and statistical analysis was performed using SPSS 30.0 software (IBM Corp., USA). Categorical variables (e.g., sex) were expressed as percentages and compared with the chi-square test. Continuous variables (e.g., ESR, CRP) were tested for normality (Kolmogorov-Smirnov test); normally distributed data were presented as mean ± standard deviation (SD) and compared with the *t*-test. Receiver operating characteristic (ROC) curves were used to evaluate the diagnostic performance of single and combined biomarkers. A *P* value <0.05 was considered statistically significant.

## Results

### Comparison of HLA-B27, ESR, and CRP levels between groups

The positivity rate of HLA-B27 in the AS group was significantly higher than that in the healthy control group (92.50% vs. 5.00%, *P*<0.001). In addition, both ESR and CRP levels were markedly elevated in AS patients compared with controls (*P*<0.001). Detailed results are shown in Table 1.

**Table 1:** Comparison of HLA-B27, ESR, and CRP levels between AS patients and healthy controls.

Group	n	HLA-B27 positivity n (%)	ESR (mm/h)	CRP (mg/L)
AS patients	120	111 (92.50)	35.85 ± 5.17	18.21 ± 3.17
Healthy controls	100	5 (5.00)	10.41 ± 1.95	5.79 ± 1.22
* t/χ^2^*	-	167.54	64.796	66.708
* P *	-	<0.001	<0.001	<0.001

### ESR and CRP levels across different disease activity states

AS patients were further stratified by disease activity into mild (n = 40), moderate (n = 50), and severe (n = 30) groups based on BASDAI scores. ESR and CRP levels increased progressively with higher disease activity, and differences across the three groups were statistically significant (\(P < 0.001 \)). Detailed results are provided in Table 2.

**Table 2:** ESR and CRP levels in AS patients with different BASDAI-defined disease activity.

Group	n	BASDAI score	ESR (mm/h)	CRP (mg/L)
Mild	40	2.74 ± 0.46	23.52 ± 3.78	10.13 ± 1.16
Moderate	50	5.68 ± 0.54	35.77 ± 4.24	21.35 ± 2.44
Severe	30	8.38 ± 0.72	46.29 ± 5.16	28.85 ± 3.27
* F *	-	-	39.012	36.528
* P *	-	-	<0.001	<0.001

### Diagnostic performance of single and combined biomarker detection

**Figure 1:**
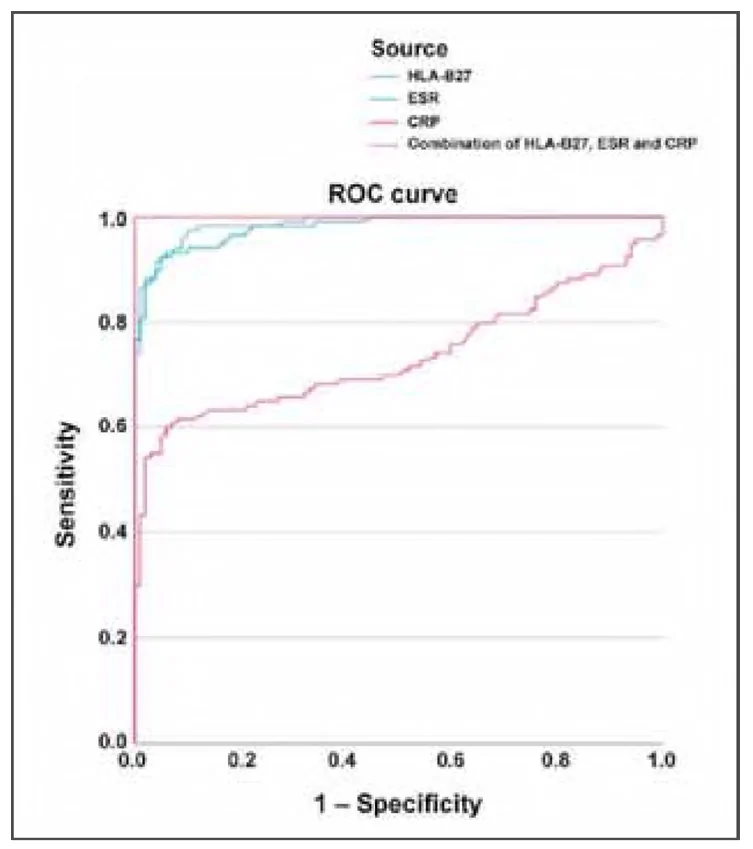
ROC curves for HLA-B27, ESR, CRP and their combination in the diagnosis of ankylosing spondylitis.

Receiver operating characteristic (ROC) curve analysis was conducted to evaluate the diagnostic efficacy of HLA-B27, ESR, and CRP, both individually and in combination. The area under the curve (AUC) for combined detection reached 0.875, significantly higher than for any single biomarker (*P*<0.05). Sensitivity and specificity values also improved markedly when combining the three markers. The detailed performance metrics are summarized in Table 3, and ROC curves are illustrated in Figure 1.

**Table 3:** Diagnostic efficacy of single versus combined detection of HLA-B27, ESR, and CRP in AS.

Biomarker(s)	AUC	Sensitivity (%)	Specificity (%)	Cut-off value	Youden index	95% CI	P
HLA-B27	0.842	81.50	88.00	1	0.592	0.901-0.974	<0.001
ESR	0.781	74.32	76.00	22.15	0.583	0.824-0.925	<0.001
CRP	0.736	67.55	73.00	15.17	0.627	0.912-0.957	<0.001
Combined	0.875	97.16	94.33	-	-	0.903-0.915	<0.001

## Discussion

AS is a chronic inflammatory disease that primarily affects the sacroiliac joints and spine, with a multifactorial etiology encompassing genetic, environmental, and immunological factors [9][10]. Among these, HLA B27 is the strongest genetic risk factor, present in over 90% of patients [11]. Nevertheless, HLA B27 positivity does not invariably lead to disease, underscoring the contribution of additional genetic and environmental influences. For example, gastrointestinal infections with organisms such as Klebsiella pneumoniae may elicit aberrant immune responses via molecular mimicry, culminating in autoinflammatory tissue damage [12]. Immunologically, dysregulated T cell activity and excessive production of cytokines such as TNF and IL 17 drive chronic inflammation, tissue injury, and fibrosis [13].

Early diagnosis is critical, as timely intervention can slow progression, preserve function, and improve quality of life [14]. However, the insidious onset and nonspecific early symptoms - such as low back pain or stiffness relieved by exercise but not rest - often delay diagnosis. Accordingly, sensitive and specific laboratory markers are essential for screening and early recognition [15][16].

HLA B27 as a diagnostic marker. Recent mechanistic studies indicate that HLA B27 misfolding induces endoplasmic reticulum stress and activates the unfolded protein response, thereby promoting pro inflammatory cytokine production [6]. In our cohort, HLA B27 positivity was 92.5%, consistent with prior reports (90-96%). While highly suggestive, HLA B27 alone is not definitive: 4-10% of healthy individuals test positive, and 5-10% of patients with AS may be negative, potentially due to other genetic (e.g., ERAP1, IL23R) or epigenetic factors. Clinically, HLA B27 testing is useful in suspected AS with atypical imaging findings, in anticipating more severe disease trajectories, and in distinguishing inflammatory from mechanical back pain [17]. Importantly, racial and ethnic differences in HLA B27 prevalence highlight the need for population specific interpretive thresholds.

Biochemical markers ESR and CRP: As acute-phase reactants, ESR and CRP are key indicators of systemic inflammation. ESR elevation reflects increased plasma fibrinogen and immunoglobulin levels [18], while CRP synthesized by the liver under IL-6 regulation, is a sensitive marker of inflammatory activity. In our study, ESR and CRP levels were significantly higher in AS patients compared with controls, and both correlated positively with disease activity as assessed by BASDAI. Patients with severe disease showed the highest ESR and CRP levels, confirming their role in disease monitoring.

Combined diagnostic approach: ROC analysis demonstrated that combining HLA-B27 with ESR and CRP substantially improved diagnostic accuracy (AUC = 0.875; sensitivity 97.2%; specificity 93.3%), significantly outperforming single-parameter testing. This synergistic effect arises because the three markers represent distinct but complementary aspects of AS pathophysiology: HLA-B27 reflects genetic susceptibility, whereas ESR and CRP indicate ongoing inflammation. Clinically, this combined strategy enhances early diagnosis, improves differentiation from other spondyloarthropathies such as psoriatic arthritis and reactive arthritis, and provides guidance for treatment planning.

Clinical utility beyond diagnosis: The integration of these biomarkers is also valuable in treatment and prognosis. Elevated ESR/CRP levels prior to therapy may indicate the need for more aggressive anti-inflammatory strategies, including biologics [19]. During therapy, dynamic decreases in ESR and CRP reflect treatment response, whereas persistently high levels suggest inadequate control and worse prognosis. In contrast, HLA-B27 remains stable over time, consistent with its role as a genetic marker rather than a disease activity marker.

Limitations: This study is constrained by its relatively small sample size and single-center design, which may limit the generalizability of the findings. In addition, the absence of other spondyloarthropathies as disease controls restricts assessment of the model's differential diagnostic performance. Future multicenter studies with larger and more diverse cohorts are warranted to validate and extend these results.

## Conclusion

Combined detection of HLA-B27, ESR, and CRP represents a robust laboratory-based diagnostic strategy for ankylosing spondylitis. By integrating immunogenetic and biochemical markers, this approach enhances diagnostic accuracy, supports treatment monitoring, and provides prognostic insights, underscoring its potential for routine application in clinical laboratory practice.

## Dodatak

### Funding

This research was funded by Beijing Jishuitan Research Funding (QN202405, to Muchuan Wang).

### Conflict of interest statement

All the authors declare that they have no conflict of interest in this work.
